# Supplementary figures and images for: The evolution of aquaculture in the Mediterranean region: An anthropogenic climax stage?
Source: PLoS One. 2024 Aug 15;19(8):e0290870. doi: 10.1371/journal.pone.0290870 (PMC11326620; doi:10.1371/journal.pone.0290870)

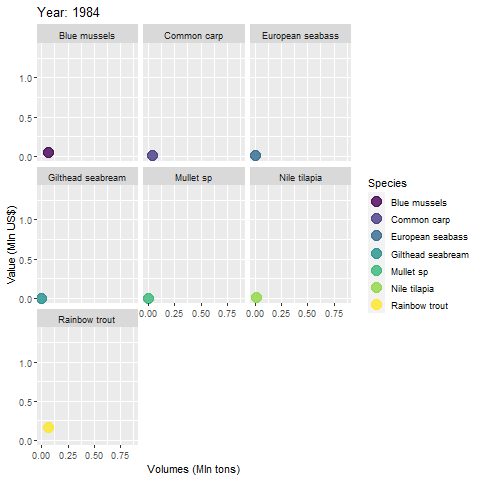

Supplement: S1 Video — (GIF) [file pone.0290870.s001.gif]
