# Supplementary material for: The evolution of aquaculture in the Mediterranean region: An anthropogenic climax stage?
Source: PLoS One. 2024 Aug 15;19(8):e0290870. doi: 10.1371/journal.pone.0290870 (PMC11326620; doi:10.1371/journal.pone.0290870)
Supplement: Attachment — Submitted filename: Mediterranean aquaculture - Response to Reviewers.docx [file pone.0290870.s002.docx]

5. Review Comments to the Author

Please use the space provided to explain your answers to the questions above. You may also include additional comments for the author, including concerns about dual publication, research ethics, or publication ethics. (Please upload your review as an attachment if it exceeds 20,000 characters)

Reviewer #1: Great amount of effort on collecting and analyzing all these data for the Mediterranean aquaculture.

Thank you very much for your comments.

I would suggest some minor corrections/ amendments:
line 43 please change the order of the word "here" with the word "analyzed"

productions [23], but the 67 years of production data analyzed here represent the entire history of

line 58 please delete the dot after 2017

OK

Reference 71 is missing so update the reference numbers

I added citation 71 in the text

In figures 1.1, 1.2, 1.5.1, 12.2 please include at the caption what is the dot line in the figure

I corrected figure 1.1 legend as suggested:

Dotted line: polynomial regression line, with equation and regression coefficients.

I uploaded most recent aquaculture data until 2020. I added economic value of aquaculture from 1984 to 2020 (FAO data on economic value are only recorded from 1984)

Fig. 1.1. a) Mediterranean aquaculture productions and annual growth rate from 1950 to 2020. b) Mediterranean aquaculture economic value and annual growth rate from 1984 to 2020. Dotted line: polynomial regression line, with equation and regression coefficients. (FAO. 2022. Fishery and Aquaculture Statistics. Global aquaculture production 1950-2020 (FishStatJ). In: FAO Fisheries and Aquaculture Division [online]. Rome. Updated 2022. ww.fao.org/fishery/statistics/software/fishstatj/en)

In figure 1.2: dotted line, legend correct as follows:

Fig 1.2 Fishery and aquaculture productions in the Mediterranean. Dotted line on the top: polynomial and linear regression lines, with relative equations and regression coefficients.

In figure 1.5.1: dotted line, legend correct as follows:

Dotted lines: polynomial regression lines, with relative equations and regression coefficients.

In figure 1.2 please explain in the caption what is the black line

Fig 1.2 Fishery and aquaculture productions in the Mediterranean. Black line, bottom: ratio aquaculture/fishery volumes. Dotted line, top: polynomial and linear regression lines, with relative equations and regression coefficients.

In figures 2.3 and 2.4 please explain in the caption what is the black and the blue lines

Fig 2.3 – Aquaculture value (blue line) and GDP pro capita (black line) (considering the 3 previous zones in aquaculture zonation obtained in fig 2.1). In the corner, on the left, the Pearson correlation coefficient (r).

Fig 2.4 – Aquaculture production (blue line) and sea food* consumption pro capita (kg/year) (black line) (considering the 3 previous zones in aquaculture zonation obtained in fig 2.1). In the corner, on the left, the Pearson correlation coefficient (r). (*sea food includes: cephalopods, crustaceans, demersal fish, fish body oil, fish liver oil, freshwater fish, marine fish, other mollusks, other pelagic fish, according all the items contained in the database: <https://www.fao.org/faostat/en/#data/CL>)

In figure 2.7 please change the font size of the legends in the figures because it is rather difficult to read them as it is now. Additionally, describe in the caption what represents the black line in the figure.

Fig 2.7 Aquaculture and fishery production in Malta, Egypt and Israel from 1950 to 2020. Black lines are the aquaculture/fishery volume ratio

I increased font size, I included description of black line. I also downloaded data until 2020 and updated the figures

Finally, in the methodology, I would suggest to give a bit more information about the selected statistical tools of the data analysis.

I inserted some short sentences in the section, to clarify the statistical methods, as follows:

Both multivariate and regression methods have been used for data elaboration.

Dendrograms have been calculated only on the rows that are fish species, as the focus of the data analysis was the study of temporal succession of farmed species in the Mediterranean. Multiple correlation plot has been obtained with R package "Performance Analytics", ". In the multiple correlation plots, frequency histograms have been added.

Reviewer #2: Review about manuscript. “The evolution of aquaculture in the Mediterranean region: an anthropogenic climax stage?”

The author presents a comprehensive analysis of production data and statistics on aquaculture in the Mediterranean. This provides new insights on what could be the evolution of the sector.

There are, however, some very important points to improve in the analysis presented here. Most of them are related to methodological aspects. Among them:

Dear Reviever #2,

Thank you for your comments, I will try to reply them as best as I can.

i) The author should better clarify and specify in Materials and Methods what is meant by the term Mediterranean region in this work. Which statistics and countries are considered under this term.

In this study I utilized all the countries of Mediterranean Sea represented in FAOSTAT databases: Malta, Egypt, Israel, Greece, Cyprus, Italy, Turkey, Croatia, Albania, France, Spain, Tunisia, Lebanon, Bosnia, Slovenia, Montenegro, Syria, Morocco, Algeria, Libya, and Palestine (indicated in tab. 2.1), thus respecting most recent geopolitical situation (in 2017).

I have excluded non-Mediterranean regions of countries which coastlines are not exclusively Mediterranean (*i.e.* Spain, France, Morocco, Egypt and Turkey) as indicated in legend of tab 2.2

I have also considered socio-political changes occurred in the course of 68 years analyzed, in particular for countries corresponding to ex-Yugoslavia (as indicated in figure 2.2)

It is clear that continental aquaculture (trout) is considered, but not if the statistics include aquaculture in the Atlantic (oysters, mussels, turbot, etc.)

I have considered all the species farmed in all the considered countries during the 68 years contained in FAO databases

or in the Black Sea.
I did not consider Black Sea, I focused my analysis on Mediterranean Sea

ii) It is striking that the analysis of FAO statistics stops at the year 2017, when from March 2022 there is the dataset with 3 more years statistics (up to 2020) is availabe. This update is essential, as there have been significant variations in recent years, which could have a major impact on the trends.

I uploaded the most recent FAO databases of aquaculture productions and capture fishery (FAO. 2022. Fishery and Aquaculture Statistics. Global capture production 1950-2020 (FishStatJ). In: FAO Fisheries and Aquaculture Division [online]. Rome. Updated 2022. www.fao.org/fishery/statistics/software/fishstatj/en) and I consequently uploaded figures with the most recent available data.

During the second elaboration of data from 1950 to 2020, I also found 2 mistakes I did in the previous data elaboration in the figures 1.3 and 2.6. I corrected them and I included a correct interpretation.

tons

growth rate

|  | growth rate (%) | production |
| --- | --- | --- |
| 1950 |  | 111770 |
| 1951 | 10.6827% | 123710 |
| 1952 | -17.7932% | 101698 |
| 1953 | 24.4341% | 126547 |
| 1954 | -22.2605% | 98377 |
| 1955 | 38.3169% | 136072 |
| 1956 | -10.7568% | 121435 |
| 1957 | 1.09935% | 122770 |
| 1958 | -9.67337% | 110894 |
| 1959 | 17.6772% | 130497 |
| 1960 | 3.03302% | 134455 |
| 1961 | 7.91715% | 145100 |
| 1962 | 27.9848% | 185706 |
| 1963 | 7.72565% | 200053 |
| 1964 | -3.62304% | 192805 |
| 1965 | 8.41835% | 209036 |
| 1966 | -4.87141% | 198853 |
| 1967 | 0.709066% | 200263 |
| 1968 | 8.05641% | 216397 |
| 1969 | 11.6832% | 241679 |
| 1970 | 28.9177% | 311567 |
| 1971 | -7.07007% | 289539 |
| 1972 | 20.0505% | 347593 |
| 1973 | 0.267554% | 348523 |
| 1974 | 7.9923% | 376378 |
| 1975 | 8.47605% | 408280 |
| 1976 | 2.47012% | 418365 |
| 1977 | 11.0258% | 464493 |
| 1978 | 6.57771% | 495046 |
| 1979 | 0.795482% | 498984 |
| 1980 | 4.63542% | 522114 |
| 1981 | 2.73733% | 536406 |
| 1982 | 2.03372% | 547315 |
| 1983 | 2.09422% | 558777 |
| 1984 | 4.55334% | 584220 |
| 1985 | 13.4009% | 662511 |
| 1986 | 3.87148% | 688160 |
| 1987 | -0.461811% | 684982 |
| 1988 | 4.29223% | 714383 |
| 1989 | -5.92623% | 672047 |
| 1990 | 6.51562% | 715835 |
| 1991 | 4.56586% | 748519 |
| 1992 | -4.72092% | 713182 |
| 1993 | -2.4744% | 695535 |
| 1994 | 11.4423% | 775120 |
| 1995 | 13.5066% | 879812 |
| 1996 | 3.1126% | 907197 |
| 1997 | 3.63152% | 940142 |
| 1998 | 15.8403% | 1089063 |
| 1999 | 11.0892% | 1209831 |
| 2000 | 11.7854% | 1352414 |
| 2001 | -1.24378% | 1335593 |
| 2002 | -4.85215% | 1270788 |
| 2003 | 8.90762% | 1383985 |
| 2004 | 0.359758% | 1388964 |
| 2005 | 6.79744% | 1483378 |
| 2006 | 9.4895% | 1624143 |
| 2007 | 2.94101% | 1671909 |
| 2008 | 0.395064% | 1678515 |
| 2009 | 3.01965% | 1729200 |
| 2010 | 9.79373% | 1898553 |
| 2011 | 5.05049% | 1994439 |
| 2012 | 1.57845% | 2025920 |
| 2013 | 3.43689% | 2095549 |
| 2014 | 3.58683% | 2170713 |
| 2015 | 2.03406% | 2214866 |
| 2016 | 11.4434% | 2468323 |
| 2017 | 4.22414% | 2572588 |
| 2018 | 10.3303% | 2838345 |
| 2019 | 1.79181% | 2889203 |
| 2020 | -1.40434% | 2848629 |

Fig. 1.1a Mediterranean aquaculture productions and annual growth rate from 1950 to 2020. (FAO. 2022. Fishery and Aquaculture Statistics. Global aquaculture production 1950-2020 (FishStatJ). In: FAO Fisheries and Aquaculture Division [online]. Rome. Updated 2022. www.fao.org/fishery/statistics/software/fishstatj/en)

I uploaded the most recent FAO databases of aquaculture economic value, that starts from 1984

billion US$

growth rate (%)

|  | growth rate | Aquaculture value |
| --- | --- | --- |
| 1984 |  | 0.662962 |
| 1985 | 9.73595% | 0.727508 |
| 1986 | 33.8395% | 0.973693 |
| 1987 | 10.0809% | 1.07185 |
| 1988 | 7.95869% | 1.15716 |
| 1989 | 6.08572% | 1.22758 |
| 1990 | 18.694% | 1.45706 |
| 1991 | 2.33138% | 1.49103 |
| 1992 | 6.80725% | 1.59253 |
| 1993 | -7.24558% | 1.47714 |
| 1994 | 10.1829% | 1.62756 |
| 1995 | 13.2262% | 1.84282 |
| 1996 | 5.83544% | 1.95036 |
| 1997 | 5.75988% | 2.0627 |
| 1998 | 12.632% | 2.32325 |
| 1999 | 2.64766% | 2.38477 |
| 2000 | 12.9283% | 2.69308 |
| 2001 | -4.27015% | 2.57808 |
| 2002 | -9.34644% | 2.33712 |
| 2003 | 20.7586% | 2.82227 |
| 2004 | 8.30749% | 3.05673 |
| 2005 | 18.6419% | 3.62657 |
| 2006 | 10.3088% | 4.00042 |
| 2007 | 18.9223% | 4.7574 |
| 2008 | 6.44825% | 5.06416 |
| 2009 | 0.593655% | 5.09423 |
| 2010 | 3.54603% | 5.27487 |
| 2011 | 13.4556% | 5.98463 |
| 2012 | -2.3666% | 5.843 |
| 2013 | 4.81974% | 6.12462 |
| 2014 | 3.75131% | 6.35437 |
| 2015 | -7.34194% | 5.88784 |
| 2016 | 12.3592% | 6.61553 |
| 2017 | -6.30435% | 6.19846 |
| 2018 | 8.21348% | 6.70757 |
| 2019 | 11.4776% | 7.47744 |
| 2020 | 8.73165% | 8.13034 |

Fig 1.1b Mediterranean aquaculture economic value and annual growth rate from 1984 to 2020 (FAO. 2022. Fishery and Aquaculture Statistics. Global aquaculture production 1950-2020 (FishStatJ). In: FAO Fisheries and Aquaculture Division [online]. Rome. Updated 2022. www.fao.org/fishery/statistics/software/fishstatj/en)

Fig 1.2 Fishery and aquaculture productions in the Mediterranean

Figure 1.2.2 Evolution of two main aquaculture sectors: marine and freshwater aquaculture. Trend and annual growth rate

Fig 1.2.3 Fish and bivalve farming in the Mediterranean region. Trend and growth rate

Fig. 1.2.4 Aquaculture productions of Nile tilapia, mullets, European sea bass and gilthead seabream.

Fishery (tons)

| Aquaculture (tons) | Fishery |
| --- | --- |
| 9370 | 524330 |
| 9810 | 549334 |
| 9998 | 579968 |
| 11447 | 568997 |
| 13177 | 598086 |
| 17172 | 594889 |
| 17635 | 660163 |
| 18970 | 649580 |
| 19294 | 660360 |
| 23697 | 640840 |
| 26055 | 623494 |
| 31200 | 662770 |
| 33506 | 622848 |
| 40353 | 721109 |
| 44705 | 802302 |
| 48936 | 785182 |
| 45149 | 782817 |
| 53953 | 844731 |
| 53381 | 794309 |
| 54157 | 838322 |
| 66539 | 855520 |
| 82504 | 851593 |
| 83944 | 909295 |
| 88060 | 909954 |
| 98290 | 934456 |
| 105976 | 956530 |
| 116149 | 1014522 |
| 124970 | 990902 |
| 131714 | 1103460 |
| 143411 | 1271312 |
| 153767 | 1364492 |
| 168980 | 1461266 |
| 174451 | 1550888 |
| 181371 | 1599510 |
| 191920 | 1647933 |
| 225182 | 1701357 |
| 241855 | 1700865 |
| 251212 | 1755311 |
| 286851 | 1823653 |
| 312346 | 1569251 |
| 349946 | 1486038 |
| 377318 | 1514886 |
| 392486 | 1663355 |
| 400970 | 1783186 |
| 431365 | 1893600 |
| 492595 | 1953495 |
| 511678 | 1791555 |
| 540894 | 1723328 |
| 630161 | 1699440 |
| 744665 | 1793054 |
| 896199 | 1743772 |
| 912618 | 1818801 |
| 896002 | 1799389 |
| 1007124 | 1741868 |
| 973610 | 1739374 |
| 1134998 | 1629929 |
| 1213268 | 1840712 |
| 1276475 | 1891864 |
| 1309814 | 1663313 |
| 1343104 | 1661151 |
| 1556585 | 1608175 |
| 1634579 | 1613732 |
| 1669087 | 1478662 |
| 1781495 | 1431092 |
| 1812978 | 1330194 |
| 1862419 | 1463365 |
| 2115350 | 1367535 |
| 2189170 | 1427506 |
| 2401156 | 1416124 |
| 2501828 | 1539329 |
| 2489481 | 1381538 |

Fig 1.3 Capture fishery and aquaculture annual productions (considering only Mediterranean coastline of respective countries)

I apologize. I did a mistake in the previous data as I did not exclude fishery data from the extra Mediterranean coasts. Part of the Morocco, Spain France, Turkey and Egypt coasts are in Atlantic and Pacific oceans. Consequently, the fishery production resulted higher than production solely relative to Mediterranean Sea. However, the interpretation of data is comparable with the previous one.

Fig 1.4.1 – Heatmaps of main aquaculture species in the Mediterranean (A: original data; B: scaled data). Data scaling: x scaled = (x-µ)/s.d. (µ=mean; s.d.=standard deviation)

Fig. 1.4.2 Comparison between succession of farmed species in the Mediterranean in 3 stages (A and C) and a forest succession in 6 stages (B) (from Wikipedia https://commons.wikimedia.org/w/index.php?curid=13274746, modified)

Fig. 1.5.1 Aquaculture diversity in the Mediterranean region, considering its main components: main, relevant and minor species. A: Pareto Index trend (ratio main/relevant species); B: evolution of different components

number of species

H index

|  | richness of species | H |
| --- | --- | --- |
| 1950 | 8 | 1.23359 |
| 1951 | 8 | 1.18403 |
| 1952 | 8 | 1.11632 |
| 1953 | 8 | 1.11392 |
| 1954 | 8 | 1.12985 |
| 1955 | 9 | 1.19059 |
| 1956 | 9 | 1.22177 |
| 1957 | 9 | 1.24469 |
| 1958 | 9 | 1.26476 |
| 1959 | 9 | 1.41785 |
| 1960 | 9 | 1.44611 |
| 1961 | 9 | 1.46087 |
| 1962 | 9 | 1.49313 |
| 1963 | 9 | 1.54579 |
| 1964 | 11 | 1.55551 |
| 1965 | 11 | 1.52513 |
| 1966 | 11 | 1.44766 |
| 1967 | 11 | 1.51461 |
| 1968 | 11 | 1.46469 |
| 1969 | 11 | 1.50215 |
| 1970 | 12 | 1.51023 |
| 1971 | 12 | 1.51348 |
| 1972 | 12 | 1.53204 |
| 1973 | 13 | 1.54654 |
| 1974 | 14 | 1.57107 |
| 1975 | 14 | 1.56009 |
| 1976 | 14 | 1.61258 |
| 1977 | 14 | 1.65516 |
| 1978 | 14 | 1.61291 |
| 1979 | 15 | 1.61737 |
| 1980 | 18 | 1.6126 |
| 1981 | 18 | 1.59699 |
| 1982 | 18 | 1.63446 |
| 1983 | 18 | 1.6275 |
| 1984 | 23 | 1.63609 |
| 1985 | 33 | 1.73519 |
| 1986 | 38 | 1.78424 |
| 1987 | 41 | 1.79303 |
| 1988 | 41 | 1.81126 |
| 1989 | 43 | 1.87388 |
| 1990 | 52 | 1.9351 |
| 1991 | 49 | 1.99561 |
| 1992 | 54 | 2.00873 |
| 1993 | 49 | 1.99088 |
| 1994 | 49 | 2.06737 |
| 1995 | 55 | 2.08619 |
| 1996 | 63 | 2.1472 |
| 1997 | 65 | 2.13627 |
| 1998 | 60 | 2.20119 |
| 1999 | 64 | 2.22633 |
| 2000 | 62 | 2.19702 |
| 2001 | 62 | 2.18501 |
| 2002 | 67 | 2.18375 |
| 2003 | 60 | 2.15526 |
| 2004 | 62 | 2.15997 |
| 2005 | 66 | 2.23995 |
| 2006 | 65 | 2.19645 |
| 2007 | 70 | 2.17273 |
| 2008 | 81 | 2.07041 |
| 2009 | 81 | 2.07492 |
| 2010 | 87 | 2.01517 |
| 2011 | 89 | 1.97051 |
| 2012 | 82 | 1.75403 |
| 2013 | 80 | 1.94031 |
| 2014 | 90 | 1.87882 |
| 2015 | 84 | 1.94993 |
| 2016 | 83 | 2.04276 |
| 2017 | 86 | 1.99934 |
| 2018 | 89 | 2.01008 |
| 2019 | 93 | 1.99664 |
| 2020 | 86 | 2.07585 |

Fig 1.5.2 Aquaculture diversity in the Mediterranean region. Shannon Index

Fig. 2.2 Map aquaculture persistence (years of farming in each country) *countries corresponding to ex-Yugoslavia have been counted considering the historic change of national boundaries

number of countries

Pareto Index (%)

|  | N_80% | total countrie | Pareto Index |
| --- | --- | --- | --- |
| 1950 | 1 | 5 | 20% |
| 1951 | 1 | 5 | 20% |
| 1952 | 1 | 5 | 20% |
| 1953 | 1 | 5 | 20% |
| 1954 | 1 | 6 | 16.6667% |
| 1955 | 1 | 6 | 16.6667% |
| 1956 | 1 | 6 | 16.6667% |
| 1957 | 1 | 6 | 16.6667% |
| 1958 | 1 | 6 | 16.6667% |
| 1959 | 1 | 6 | 16.6667% |
| 1960 | 1 | 6 | 16.6667% |
| 1961 | 1 | 6 | 16.6667% |
| 1962 | 2 | 5 | 40% |
| 1963 | 2 | 5 | 40% |
| 1964 | 2 | 5 | 40% |
| 1965 | 2 | 5 | 40% |
| 1966 | 2 | 6 | 33.3333% |
| 1967 | 2 | 6 | 33.3333% |
| 1968 | 2 | 7 | 28.5714% |
| 1969 | 2 | 7 | 28.5714% |
| 1970 | 2 | 7 | 28.5714% |
| 1971 | 2 | 7 | 28.5714% |
| 1972 | 2 | 7 | 28.5714% |
| 1973 | 2 | 8 | 25% |
| 1974 | 2 | 9 | 22.2222% |
| 1975 | 2 | 11 | 18.1818% |
| 1976 | 2 | 11 | 18.1818% |
| 1977 | 2 | 11 | 18.1818% |
| 1978 | 2 | 11 | 18.1818% |
| 1979 | 2 | 11 | 18.1818% |
| 1980 | 2 | 11 | 18.1818% |
| 1981 | 2 | 11 | 18.1818% |
| 1982 | 2 | 11 | 18.1818% |
| 1983 | 2 | 12 | 16.6667% |
| 1984 | 2 | 12 | 16.6667% |
| 1985 | 3 | 11 | 27.2727% |
| 1986 | 3 | 11 | 27.2727% |
| 1987 | 3 | 12 | 25% |
| 1988 | 3 | 12 | 25% |
| 1989 | 3 | 12 | 25% |
| 1990 | 3 | 13 | 23.0769% |
| 1991 | 3 | 13 | 23.0769% |
| 1992 | 3 | 15 | 20% |
| 1993 | 3 | 15 | 20% |
| 1994 | 3 | 15 | 20% |
| 1995 | 3 | 15 | 20% |
| 1996 | 3 | 15 | 20% |
| 1997 | 3 | 15 | 20% |
| 1998 | 4 | 14 | 28.5714% |
| 1999 | 4 | 14 | 28.5714% |
| 2000 | 4 | 14 | 28.5714% |
| 2001 | 4 | 14 | 28.5714% |
| 2002 | 4 | 15 | 26.6667% |
| 2003 | 4 | 15 | 26.6667% |
| 2004 | 4 | 15 | 26.6667% |
| 2005 | 4 | 15 | 26.6667% |
| 2006 | 4 | 16 | 25% |
| 2007 | 4 | 16 | 25% |
| 2008 | 4 | 16 | 25% |
| 2009 | 4 | 16 | 25% |
| 2010 | 4 | 16 | 25% |
| 2011 | 4 | 16 | 25% |
| 2012 | 4 | 16 | 25% |
| 2013 | 4 | 16 | 25% |
| 2014 | 3 | 17 | 17.6471% |
| 2015 | 3 | 17 | 17.6471% |
| 2016 | 3 | 17 | 17.6471% |
| 2017 | 3 | 17 | 17.6471% |
| 2018 | 3 | 17 | 17.6471% |
| 2019 | 3 | 17 | 17.6471% |
| 2020 | 3 | 17 | 17.6471% |

Fig. 2.6 Geographical expansion of aquaculture in the Mediterranean region. In red the number of countries producing the 80% of annual production. Pareto index (%) = (number of countries producing the 80% of annual production/total countries aquaculture producing)

I apologize, in this figure I fond an error I have done in the previous data elaboration. Pareto index is 23% for the entire considered period, even if in the first years there are some oscillation caused by low number of countries producing the 80% of total production.

This value is closer to that relative to species diversity (Fig. 1.5.1) and also with a previous study on world aquaculture diversity (Sicuro B. World aquaculture diversity: origins and perspectives. Rev. Aquac. 2021; 13, 1619-1634 https://doi.org/10.1111/raq.12537).

Fig 2.7 Aquaculture and fishery production in Malta, Egypt and Israel from 1950 to 2020

iii) Another aspect is that it is highly advisable to carry out an analysis of production in the Mediterranean considering the value of production. This would help the reader to gain knowledge on the reasons being the development of the sector. For example, while in Egypt, the leading country in the Mediterranean, the main driving force for development is the need to provide animal protein of high nutritional value and affordable prices (tilapia, mullets), in the rest of the Mediterranean that is different, and thus higher value species are produced.

I added the evolution of economic value of Mediterranean aquaculture from 1984 to 2020, including annual growth rate and polynomial model of regression

billion US$

growth rate (%)

|  | growth rate | Aquaculture value |
| --- | --- | --- |
| 1984 |  | 0.662962 |
| 1985 | 9.73595% | 0.727508 |
| 1986 | 33.8395% | 0.973693 |
| 1987 | 10.0809% | 1.07185 |
| 1988 | 7.95869% | 1.15716 |
| 1989 | 6.08572% | 1.22758 |
| 1990 | 18.694% | 1.45706 |
| 1991 | 2.33138% | 1.49103 |
| 1992 | 6.80725% | 1.59253 |
| 1993 | -7.24558% | 1.47714 |
| 1994 | 10.1829% | 1.62756 |
| 1995 | 13.2262% | 1.84282 |
| 1996 | 5.83544% | 1.95036 |
| 1997 | 5.75988% | 2.0627 |
| 1998 | 12.632% | 2.32325 |
| 1999 | 2.64766% | 2.38477 |
| 2000 | 12.9283% | 2.69308 |
| 2001 | -4.27015% | 2.57808 |
| 2002 | -9.34644% | 2.33712 |
| 2003 | 20.7586% | 2.82227 |
| 2004 | 8.30749% | 3.05673 |
| 2005 | 18.6419% | 3.62657 |
| 2006 | 10.3088% | 4.00042 |
| 2007 | 18.9223% | 4.7574 |
| 2008 | 6.44825% | 5.06416 |
| 2009 | 0.593655% | 5.09423 |
| 2010 | 3.54603% | 5.27487 |
| 2011 | 13.4556% | 5.98463 |
| 2012 | -2.3666% | 5.843 |
| 2013 | 4.81974% | 6.12462 |
| 2014 | 3.75131% | 6.35437 |
| 2015 | -7.34194% | 5.88784 |
| 2016 | 12.3592% | 6.61553 |
| 2017 | -6.30435% | 6.19846 |
| 2018 | 8.21348% | 6.70757 |
| 2019 | 11.4776% | 7.47744 |
| 2020 | 8.73165% | 8.13034 |

Fig 1.1b Total aquaculture from 1984 to 2020 (trend and growth rate) (FAO. 2022. Fishery and Aquaculture Statistics. Global aquaculture production 1950-2020 (FishStatJ). In: FAO Fisheries and Aquaculture Division [online]. Rome. Updated 2022. www.fao.org/fishery/statistics/software/fishstatj/en)

iv) The author analyses aquaculture in Egypt separately, which is appropriate as it is the main producer country in the region. However, it would be advisable to provide a more in-depth description of aquaculture development in this country, since a very significant amount of production (mullets) is carried out in brackish waters, and this is reflected in the statistics. In fact Egyptian mullets production may account for near 20% production in the regions.

I added following figure (Fig. 2.8), considering main species of Egyptian aquaculture

Figure 2.7b Aquaculture productions in Egypt (main species) from 1950 to 2020

v) Please, revise the sentence in lines 168 and 169. “Fish farming is the main production in the Mediterranean [53, 6, 54], in fact in 2017 accounted for the 96% of entire aquaculture production in the region.” This must be a mistake, as bivalve production may be around 20% of the whole production.

I checked data, I re-made elaborations, I introduced a figure on Egyptian aquaculture (Fig 2.7b) and I consequently correct as follows:

Fish farming is the main production in the Mediterranean [53, 6, 54], in fact in 2020 accounted for more than the 90% of entire aquaculture production in the region. Within fish species, Mediterranean aquaculture has 4 species with potential further growth: Nile tilapia, European sea bass, gilthead seabream and in less extent, mullets (Fig. 1.2.4)

Following: carps and mullets productions in Egypt (not introduced in the manuscript)

|  | Common carp | Mullets |
| --- | --- | --- |
| 1950 | 65% | 0% |
| 1953 | 55.5556% | 0% |
| 1954 | 53.3333% | 0% |
| 1955 | 50.7463% | 0% |
| 1956 | 50% | 0% |
| 1957 | 47.3684% | 0% |
| 1958 | 46.5116% | 0% |
| 1959 | 45.6522% | 0% |
| 1960 | 45.6522% | 0% |
| 1961 | 44.2308% | 0% |
| 1962 | 44.2308% | 0% |
| 1963 | 43.3333% | 0% |
| 1964 | 42.8571% | 0% |
| 1965 | 41.9355% | 0% |
| 1966 | 42.3729% | 0% |
| 1967 | 42.8571% | 0% |
| 1968 | 42.8571% | 0% |
| 1969 | 42.3729% | 0% |
| 1970 | 43.3333% | 0% |
| 1971 | 42.8571% | 0% |
| 1972 | 41.875% | 0% |
| 1973 | 42.2222% | 0% |
| 1974 | 42.2222% | 0% |
| 1975 | 42.2222% | 0% |
| 1976 | 40.9091% | 0% |
| 1977 | 40.3846% | 0% |
| 1978 | 40% | 0% |
| 1979 | 39.7059% | 0% |
| 1980 | 39.4737% | 0% |
| 1981 | 39.2857% | 0% |
| 1982 | 38.1667% | 0% |
| 1983 | 38% | 0% |
| 1984 | 44.4444% | 0% |
| 1985 | 31.0663% | 3.58457% |
| 1986 | 32.967% | 1.0989% |
| 1987 | 32.3276% | 3.01724% |
| 1988 | 36.3985% | 4.21456% |
| 1989 | 39.6694% | 9.09091% |
| 1990 | 40.3773% | 9.69055% |
| 1991 | 37.2608% | 11.8474% |
| 1992 | 39.1267% | 12.9275% |
| 1993 | 38.0492% | 13.6536% |
| 1994 | 31.7792% | 14.6211% |
| 1995 | 27.621% | 20.2256% |
| 1996 | 23.3319% | 22.0558% |
| 1997 | 8.04046% | 18.7051% |
| 1998 | 8.92466% | 20.3624% |
| 1999 | 9.87732% | 18.9976% |
| 2000 | 4.81045% | 23.6788% |
| 2001 | 5.3581% | 28.2689% |
| 2002 | 4.34073% | 30.0367% |
| 2003 | 3.82002% | 30.4615% |
| 2004 | 0% | 28.1317% |
| 2005 | 0% | 28.9841% |
| 2006 | 0% | 38.9256% |
| 2007 | 0% | 39.7326% |
| 2008 | 1.64309% | 30.1684% |
| 2009 | 1.65672% | 29.7637% |
| 2010 | 3.44949% | 12.6175% |
| 2011 | 3.41116% | 11.5524% |
| 2012 | 2.32525% | 12.7391% |
| 2013 | 4.2319% | 10.5828% |
| 2014 | 4.04541% | 10.522% |
| 2015 | 2.55356% | 13.3789% |
| 2016 | 3.64788% | 11.2191% |
| 2017 | 0.00167038% | 14.942% |
| 2018 | 5.00392% | 15.1842% |
| 2019 | 4.85849% | 14.8588% |
| 2020 | 4.71136% | 19.9641% |

vi) Besides the need of updating the statistics, considering the latest dataset from March 2022 (FAO-FishStatJ. Aquaculture Production (Quantities and values) 1950-2020 (Release date: March 2022), the author may profit of the occasion to include some recent relevant publications. i.e.:

Llorente, I., Fernandez-Polanco, J., Baraibar-Diez, E., Odriozola, M. D., Bjorndal, T., Asche, F., … Basurco, B. (2020). Assessment of the economic performance of the seabream and seabass aquaculture industry in the European Union. Marine Policy, 117, 103876.<https://doi.org/>10.1016/j.marpol.2020.103876

Added as 88th reference

COMMUNICATION FROM THE COMMISSION TO THE EUROPEAN PARLIAMENT, THE COUNCIL, THE EUROPEAN ECONOMIC AND SOCIAL COMMITTEE AND THE COMMITTEE OF THE REGIONS Strategic guidelines for a more sustainable and competitive EU aquaculture for the period 2021 to 2030

Brussels, 12.5.2021

Added as 82th reference

Fernandez Sanchez, José Luís & Llorente, Ignacio & Fernandez Polanco, Jose. (2023). Profitability differences in aquaculture firms of the Nordic and Mediterranean-EU regions. Aquaculture Economics & Management. 1-17. 10.1080/13657305.2022.2163721.

Best regards

6. PLOS authors have the option to publish the peer review history of their article ([what does this mean?](https://journals.plos.org/plosone/s/editorial-and-peer-review-process#loc-peer-review-history)). If published, this will include your full peer review and any attached files.


If you choose “no”, your identity will remain anonymous but your review may still be made public.


**Do you want your identity to be public for this peer review?** For information about this choice, including consent withdrawal, please see our [Privacy Policy](https://www.plos.org/privacy-policy).

Reviewer #1: No

Reviewer #2: No

[NOTE: If reviewer comments were submitted as an attachment file, they will be attached to this email and accessible via the submission site. Please log into your account, locate the manuscript record, and check for the action link "View Attachments". If this link does not appear, there are no attachment files.]

While revising your submission, please upload your figure files to the Preflight Analysis and Conversion Engine (PACE) digital diagnostic tool, <https://pacev2.apexcovantage.com/>. PACE helps ensure that figures meet PLOS requirements. To use PACE, you must first register as a user. Registration is free. Then, login and navigate to the UPLOAD tab, where you will find detailed instructions on how to use the tool. If you encounter any issues or have any questions when using PACE, please email PLOS at [figures@plos.org](mailto:figures@plos.org). Please note that Supporting Information files do not need this step.
